# Guiding role of esophageal variceal diameter in treatment of endoscopic ligation: an animal experimental study

**DOI:** 10.1038/s41598-024-53752-3

**Published:** 2024-02-16

**Authors:** Zhiqun Li, Enqiang LingHu, Weimin Li, Licai Zhou

**Affiliations:** 1 Department of Gastroenterology, Chinese PLA General Hospital, Beijing, 100853 China; 2 Department of Gastroenterology, Chinese PLA 984 Hospital, Beijing, 100094 China; 3 Beijing Friendship Hospital Pinggu Campus, Capital Medical University, Beijing, 101200 China

**Keywords:** LDRf typing, Varicose vein, Polycyclic loop ligator, Esophageal varicose ligation, Gastroenterology, Medical research

## Abstract

In this study, according to the Location, Diameter, Risk factor (LDRf) classification principle, the influence and effect of esophageal varices diameter on the degree of complete ligation of multicyclic ligator were investigated. Methods. The esophageal veins of healthy piglets were filled with methylene blue solution, and the in vitro pig esophageal varices model was made, which were divided into three groups according to the diameter of esophageal varices: D_1_, 0.4–1.0 cm; D_2_, 1.1–1.5 cm; and D_3_, 1.6–2.0 cm. Finally, the ligation effect of each group was analyzed statistically. A total of 407 ligations were performed on the simulated esophageal variceal model. There were 103 ligations in the D_1_ group and 98 were complete (95.15%, 98/103); 151 ligations in the D_2_ group and 47 were complete (31.13%, 47/151); and 153 ligations in the D_3_ group but none were complete (0%, 0/153). There was significant difference in the degree of complete ligation between the two groups (χ^2^ = 38.0014, *P* ≤ 0.001). In the varicose ligation model, the complete ligation effect was the most complete and robust when the varicose diameter was 0.4–1.0 cm. This study showed that the varicose vein diameter in LDRf classification was reasonable and feasible to guide endoscopic varicose vein ligation.

## Introduction

Esophageal variceal bleeding is a serious complication of cirrhosis. If no treatment is given after the first bleeding, the 6-week mortality rate is as high as 15 to 20%, and the 1-year rebleeding rate is as high as 60%. Band ligation or non-selective β blockers (NSBB) are currently the main methods for the primary prevention of cirrhosis and esophageal variceal bleeding. Band ligation combined with NSBB is the main method for the secondary prevention of cirrhosis and rebleeding of esophageal varices^1–5^.

Meta-analysis showed that the emergency hemostasis rate of EIS was 95% (76–100%), which was not significantly different from EVL^6^, while EVL had a higher rate of variceal eradication, lower rebleeding rate, and lower complication rate^7^. At present, foreign guidelines recommend EVL as the first choice of treatment for acute EVB^3,8–10^.

Endoscopic variceal ligation (EVL) is a safe and effective method for the eradication of esophageal varices. It is reported that the effective rate of ligation for esophageal varices is equal to or higher than sclerotherapy^11,12^. But its effect on different diameters of esophageal varices was not yet well investigated now.

For esophagogastric varices (GOV), the classification and grading criteria are different between domestic and foreign countries, the sarin typing is often used abroad^13^. In 2008, a new LDRf typing was proposed by the professor Linghu Enqiang in China^7,14^, and this consensus was recommended by Chinese Association of Digestive Endoscopy. LDRf is based on the previously published grading system^15–17^, which covers the entire gastrointestinal varicosis, and is easy to memorize and easy to write, and integrates recording, typing, treatment, and timing of treatment. The LDRf classification is described and recorded by three factors:location (L), diameter (D) and risk factor (Rf), which was expressed as follows: LXxD_0.3–5.0_ Rf_0,1,2._ (see Tables 1, 2).

**Table 1:** LDRf classification for gastrointestinal varices.

Factors	Classification
Location (L)	Le: esophageal varices
	Le_s_: superior esophagus
	Le_m_: middle esophagus
	Le_i_: inferior esophagus
	Lg: gastric varices
	Lg_f_: gastric fundus
	Lg_b_: gastric body
	Lg_a_: gastric antrum
	Leg: gastroesophageal varices (extension of esophageal varices)
	Le,Lg: co-existing and independent varices in esophagus and stomach
	Ld: duodenal varices
	Ld_1_: duodenal bulb
	Ld_2_: descending duodenum Ld_1,2_: the junction of Ld_1_ and Ld_2_ Ld_3_: horizontal duodenum Ld_2,3_: the junction of Ld_2_ and Ld_3_ Ld_4_: ascending duodenum Ld_3,4_: the junction of Ld_3_ and Ld_4_
	Lj: jejunal varices
	Li: ileac varices
	Lb: biliary varices
	Lc: colonic varices
	Lc_a_: ascending colon Lc_t_: transverse colon Lc_d_: descending colon Lc_s_: sigmoid colon
	Lr: rectal varices
Diameter (D)	D_0_: 0 cm (no varices)
	D_0.3_: ≤ 0.3 cm
	D_1_: 0.4–1.0 cm
	D_1.5_: 1.1–1.5 cm
	D_2_: 1.6–2.0 cm
	D_3_: 2.1–3.0 cm
Risk factors (Rf)	Rf_0_: RC (−); no erosion, no thrombus, no active bleeding
	Rf_1_: RC (+), HVPG > 12 mmHg; no erosion, no thrombus, no active bleeding
	Rf_2_: presence of erosion, or thrombus, or active bleeding, or freshblood excluding non-variceal bleeding

RC: red color sign; RC (−): negative red color sign; RC (+): positive red color sign; HVPG: hepatic venous pressure gradient.

**Table 2:** According to LDRf type, selection of treatment options based on the diameter grading and risk factors of the varices.

Diametergrades	Recommended treatments	Treatments not recommended
D_0.3_	APC, laser, hemostatic clips	EVL, EVS and injection of tissue adhesives
D_1.0_	EVL, EVS	APC, laser, hemostatic clips
D_1.5_	EVL, EVS	APC, laser, hemostatic clips
D_2.0_	EVS for esophageal varices. Injection of tissueadhesives for non-esophageal varices	EVL,APC, laser, hemostatic clips
D_3.0_	EVS for esophageal varices Injection of tissueadhesives forvaricesin places other thangastric cardia and esophagus	EVL,APC, laser, hemostatic clips

Risk factors	Timing and management	
Rf_0_	D_0.3_: no treatment, endoscopic follow-up every year	
RC (−)	D_1.0_:selective treatmentor endoscopic follow up every 6 months	
HVPG < 12 mmHg	D_1.5_ and above: EVS for esophageal varices and tissue adhesives for the cardicvarices Endoscopic observation every 6 months Injection of tissue adhesives for non-esophageal varices, or endoscopic observation every 3–6 months	
No erosion		
No thrombus		
No active bleeding		
Rf_1_	Treatment within 3 months	
RC (+) or HVPG > 12 mmHg		
No erosion		
No thrombus		
No active bleeding		
Rf_2_	Immediate treatment is required	
Erosion		
Thrombus		
Active bleeding		
Presence of fresh blood, excluding non-variceal bleeding		

APC, argon plasma coagulation; EVL, endoscopic variceal ligation; EVS, endoscopic varicealsclerotherapy; HVPG: hepatic venous pressure gradient; RC: red color sign; RC (−): negative red color sign; RC (+): positive red color sign.

In this study, a model of esophageal varices of pigs with different diameters was constructed as the research object, and in vitro simulated EVL experiments were carried out to observe the influence of variceal vessels of different diameters on the effect of banding.

## Materials and methods

We made model EVL of animals pig, and this simulated experimental study was carried out according to the LDRf classification criteria^15^. Institutional Animal Care and Use Committee of Capital Medical University (protocol number: 201203031-001) and PLA General Hospital (protocol number: 20110323-001) approved all procedures. All experimental procedures were performed in accordance with relevant guidelines and regulations. This study is reported in accordance with ARRIVE guidelines, https://arriveguidelines.org.

### Animals and equipment

#### Experimental animals

Experimental pigs, 3–24 months old, all female, weighing 40 ± 20 kg, were clinically healthy and were purchased from Liuli River Experimental Pig Farm, Fangshan District, Beijing.

#### Experimental equipment and drugs

Experimental equipment includes anesthesia machine, monitor, anesthesia bed, laryngoscope, stethoscope and other commonly used items. The equipment includes Olympus-240 electronic gastroscope, Wilson-Cook six-shot ligation device, Vernier caliper, and T-shape tubes. Glucose methylene blue solution was used to fill the veins for visibility.

The experimental drugs included atropine, dexamethasone, midazolam, sodomethacin, succinylcholine, diazepam injection, lidocaine, etc.

### Anesthesia method

Piglets were given intravenous general anesthesia, and the anesthesia method is divided into two stages:

#### Anesthesia induction phase

Administration before induction: Intramuscular injection of atropine 0.5 mg and dexamethasone 10 mg is routinely given before anesthesia induction to reduce the secretion of salivary glands and tracheal glands and prevent allergic reactions. The anesthesia induction drugs were midazolam 0.1–0.2 mg/kg and Sumisan 0.25–0.3 ml/kg compound drugs.

#### Anesthesia maintenance phase

The anesthesia maintenance medication is to use 6 succinylcholine, 6 diazepam injection, and 7 lidocaine to be added to 500 ml of balanced solution to prepare an anesthetic mixture for slow intravenous infusion, first rapid infusion of 5–10 ml, and after the experimental animal anesthesia is complete, the drip rate is slowed down to 40–60 drops/min.

### Selecting the pig veins

We opened the abdominal cavity of the pigs and selected the inferior vena cava, portal vein, superior mesenteric vein, and limb veins of different thickness.

### Pig variceal model and measurement of varicose vein diameter

**Figure 1:**
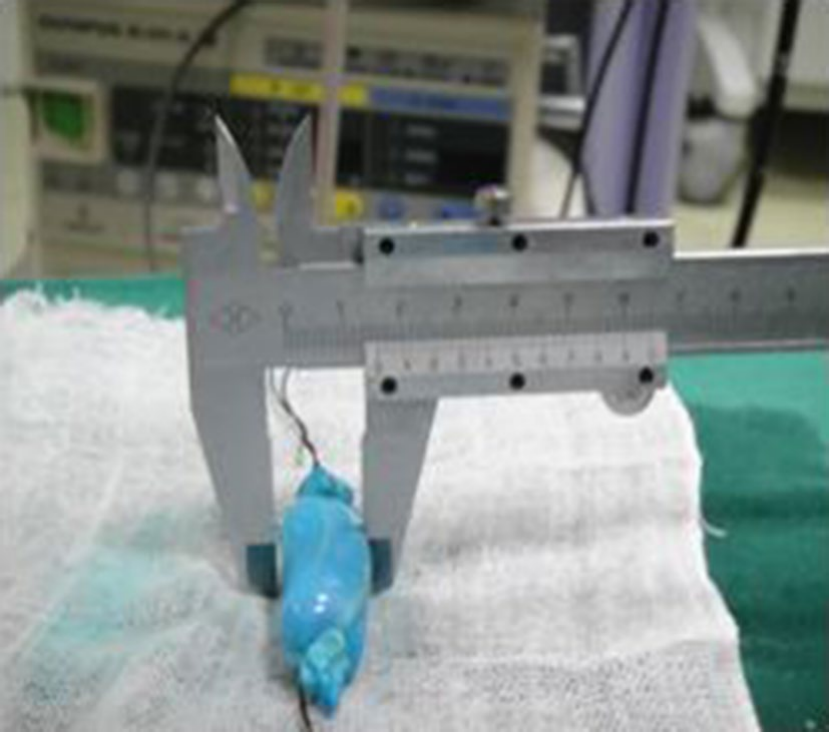
Vein with diameter of 1.6 cm.

**Figure 2:**
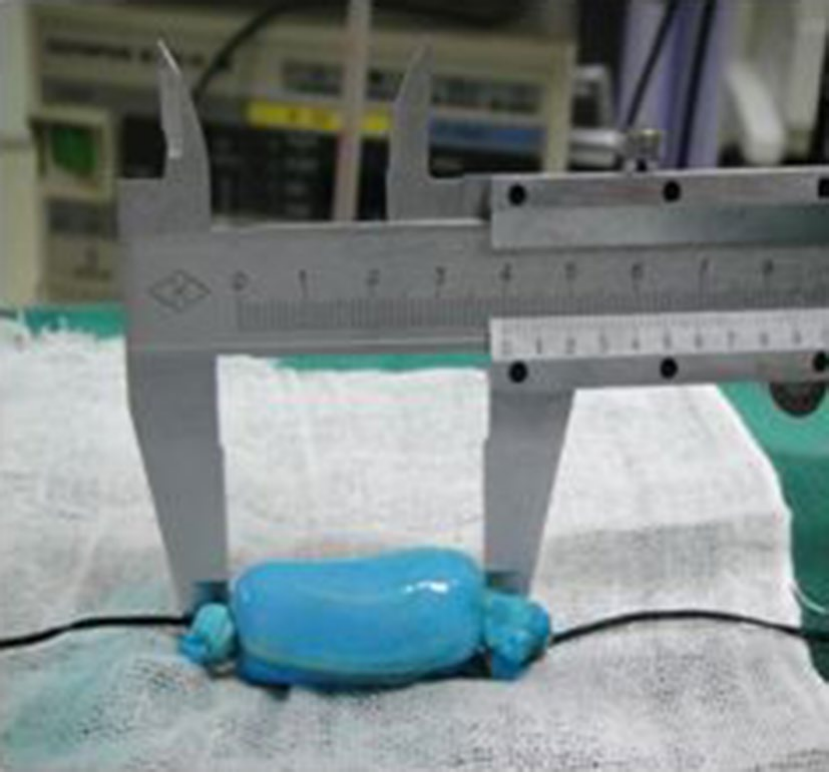
Vein with length of 4 cm.

One end of the vein was ligated and the other end connected to the T tube, whose other end was connected to the glass column burette filled with methylene blue liquid The T tube was opened so that the water flowed into the vein, and the water column gradually decreased, while the porcine vein was gradually filled. When the water flow stopped, the end of the vein that was connected to the T tube was ligated to produce porcine variceal models with different diameters ranging from 0.4 to 2.0 cm and lengths of 3.0–4.0 cm, as measured with a vernier caliper (Figs. 1, 2).

### Selecting the pig esophagus

**Figure 3:**
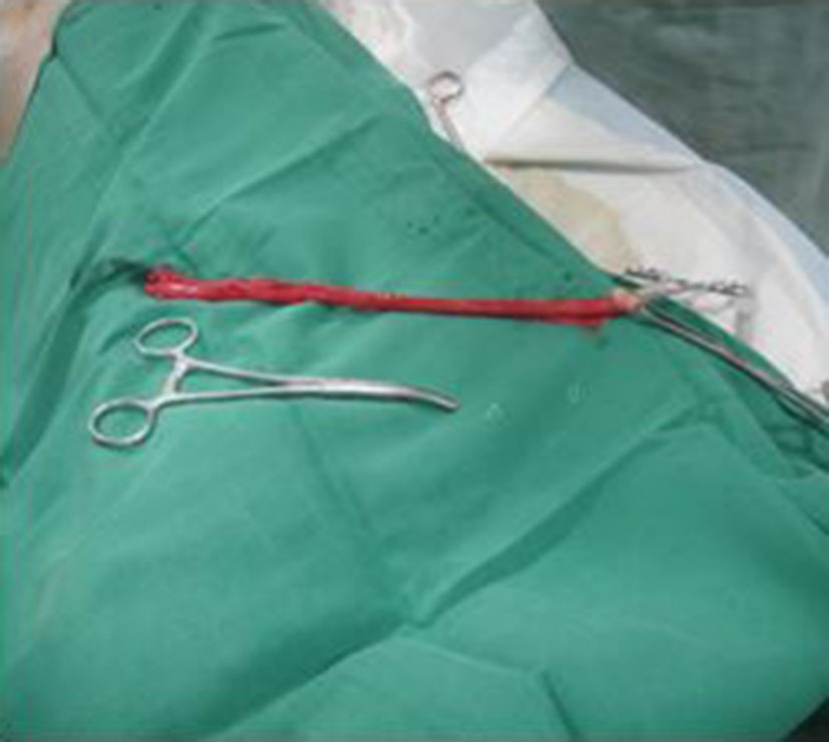
Esophagus with length of 40 cm.

**Figure 4:**
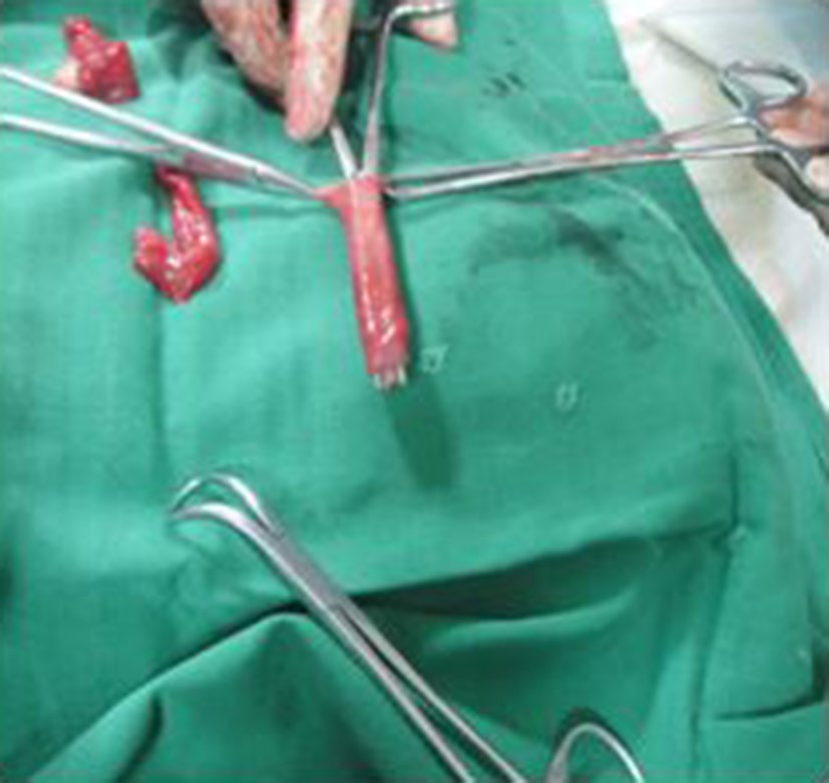
Submucosal tunnel.

The pig's esophagus is about 40 cm long, divided into three sections, each about 13 cm long,, and the esophageal lining was flipped out. The mucosa and muscularis propria layers were bluntly dissected with a hemostat, forming a submucosal tunnel (Figs. 3, 4).

### Pig model of esophageal varices

Using a hemostat, the pig modeled varicose vein was introduced through the esophageal tunnel between the muscularis propria and submucosal layers, to form the pig model of esophageal varices.

### Measuring esophageal variceal diameter

**Figure 5:**
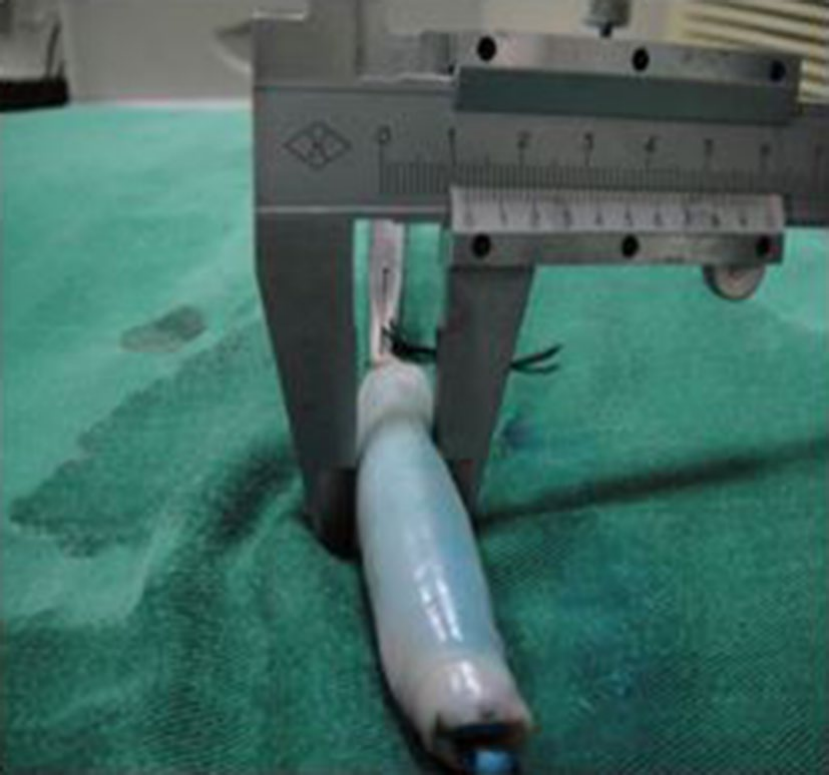
In vitro measurment of the diameter of the esophageal varices.

The diameter of the esophageal varices was measured by a vernier caliper, and divided into three groups according to the LDRf classification of the variceal different diameters: D_1_, 0.4–1.0 cm; D_2_, 1.0–1.5 cm; and D_3_, 1.6–2.0 cm (Fig. 5).

### Pig variceal ligation model

**Figure 6:**
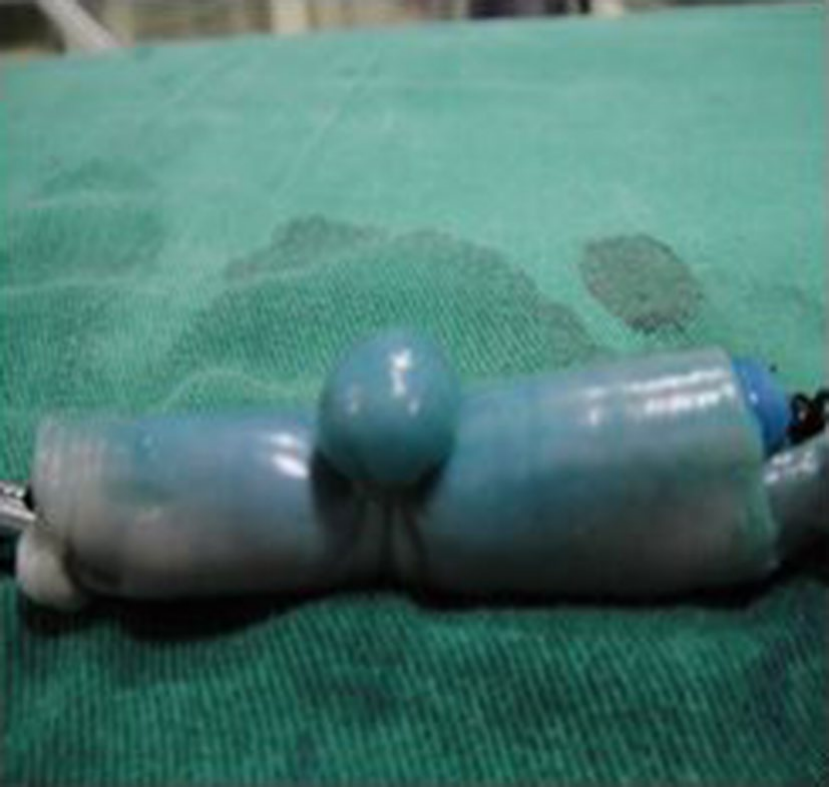
In vitro ligation of the esophageal varices.

**Figure 7:**
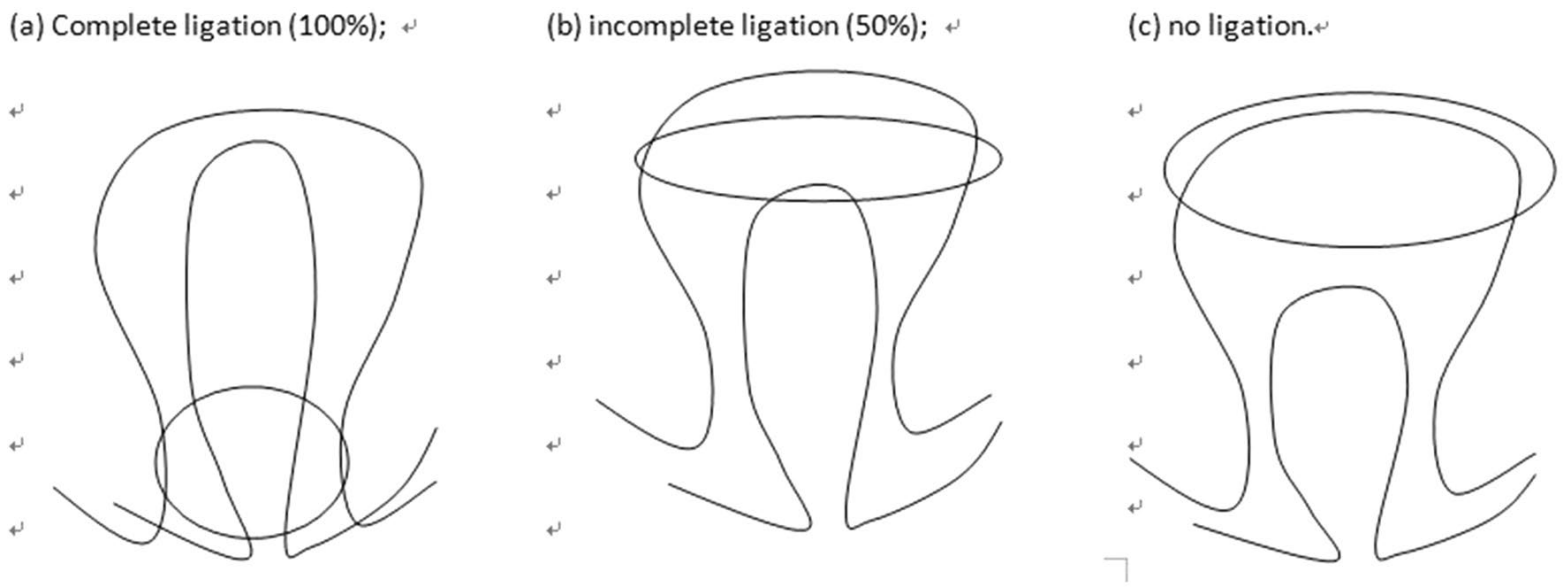
The effect of ligation pattern.

The multiple-shot ligation device pre-fixed to the front end of the endoscope was used to target the esophageal varicose vein to create continuous suction. When the suction pressure rose to 0.03–0.05 MPa^18^ and the endoscopic vision gradually became completely blue, the rubber band was released to stop the suction and ligated at the base of the vein^19^ (Fig. 6). The esophageal mucosa was cut off and separated after ligation, and the vein was dissected after submucosal ligation. The ligation effect was observed and judged as complete (100%), incomplete (50%), and no ligation (Fig. 7a–c).

### Statistical analysis

We have used SPSS Version 13.0 software to carry out the statistical analysis which is given in this manuscript. *P* < 0.05 was considered statistically significant.

## Results

### Esophageal variceal diameter

A total of 407 ligation models of simulated esophageal varices were formed: 103 in the D_1_ group, 151 in the D_2_ group, and 153 in the D_3_ group.

### Pig variceal ligation rate

Univariate analysis of the effect of in vitro ligation of esophageal varices of different diameters was as follows. For the D_1_ group, there were 98 complete ligations (24.1%, 98/407), one incomplete ligation (0.2%, 1/407), and four ligation failures (1%, 4/407). For the D_2_ group, there were 47 complete ligations (11.5%, 47/407), 41 incomplete ligations (10.1%, 41/407), and 63 ligation failures (15.5%, 63/407). For the D_3_ group, there were no complete ligations (0%, 0/407), no incomplete ligations (0%, 0/407), and 153 ligation failures. The results showed that there was a significant difference in the number of complete ligations between the groups (χ^2^ = 38.0014, *P* < 0.05). The results for ligation of varices with different diameters are shown in Tables 3 and 4.

**Table 3:** The ratio of complete ligation for the swine esophageal variceal difference diameter.

Group	Ligation cases (%)			Subtotal	Constituent ratio of ligation results (%)		
	0%	50%	100%		0%	50%	100%
D_1_	4	1	98	103	3.88	0.97	95.15
D_2_	63	41	47	151	41.72	27.15	31.13
D_3_	153	0	0	153	100.00	0.00	0.00
Total	220	42	145	407	54.05	10.32	35.63

Grouping by vein diameter: D_1_ group, 0.3–1.0 cm; D_2_ group, 1.1–1.5 cm; D_3_ group, 1.6–2.0 cm.

**Table 4:** Linear trend test for bidirectional ordered grouping data.

Source of variation	Degrees of freedom	Chi-square value	*P* value
Linear regression component	1	38.0014	0.0001
Deviation regression component	3	39.1623	0.0001
Total chi-square	4	77.1636	0.0001

Linear trend χ^2^ = 38.0014, *P* < 0.05.

## Discussion

Endoscopic treatment of esophageal varices is mainly by ligation and supplemented by sclerosis.. Ligation and sclerotherapy have their own advantages and disadvantages, and should be based on the characteristics of each method. Re-examination of gastroscopy 1 week after emergency ligation of severe esophagogastric varices and the discharge time should be determined according to the results of gastroscopy, which can effectively reduce rebleeding after banding. Severe esophageal varices should be mainly ligation, with emphasis on preventing re-bleeding and alleviating varices. Mild and moderate esophageal varices should be mainly scleroded, focusing on the treatment of deep mucosal varices and perforating branches to reduce recurrence, and at the same time, due to the small amount of sclerosing agent, the incidence of complications after sclerosing agent injection is effectively reduced. When necessary, the treatment should be supplemented with transparent caps and tissue glue.

EVL therapy is one of the latest methods in treatment of esophageal variceal bleeding, which was first reported by Stiegmann in 1986^20^. The therapeutic effect of EVL has the following mechanisms^21^: (1) mechanical blockade of the blood flow of the varicose veins to make the varices shrink; (2) thrombosis at the ligation site of the varicose vein and occurrence of organizing or calcification; (3) scar formation and fibrosis in the wall of the ligated vein; (4) degradation or disappearance of the varicose veins after ligation.

Esophageal variceal bleeding is the major complication and cause of death in patients with cirrhosis. How to seize the opportunity to reduce or eliminate the esophageal varicosis is the most important means to prevent bleeding.

The principle of EVL is to block the varicose vein blood flow emergency hemostasis, so that the ligation of venous thrombosis, tissue necrosis, fibrosis formation, and finally make the varicose vein disappear. EVL includes single ligature, multiple ligature and nylon rope ligature. Repeated ligation is superior/not inferior to repeated sclerotherapy in the treatment of esophageal varices, but the incidence of side effects (including esophageal stenosis, perforation, infection, etc.) is significantly lower. Simultaneous combination of ligation and sclerotherapy for esophageal variceal vein rupture bleeding is not superior to repeated ligation treatment^22–25^.

Many studies have shown 90% hemostasis after emergency therapy for esophageal variceal bleeding by endoscopic ligation^26^. Ligation therapy has become the first choice of endoscopic treatment with good results, due to the rapid disappearance of varices, few complications, simple operation, and low rebleeding rate after ligation. However, variceal ligation has some risk, especially the ligation ring may fall off triggering the bleeding which is difficult to control. Three results can occur after ligation of esophageal varices: (1) complete ligation, indicating that the ligation effect is certain and complete, and the ligation ring at the root of the ligated varicose veins is not easy to slip off and is strong and stable; (2) incomplete ligation (the root of the varicose veins is not completely ligated, ligation only less than or equal to 50%), indicating that the effect of banding was uncertain and incomplete, and the ligature knot was easy to fall off early, which was easy to induce massive bleeding and increase mortality. (3) ligation failure, indicating that the banding ring cannot ligature the root of varicose veins.

From above consideration, it is necessary to carry out clinical studies on the surgical methods, indications and other aspects of EVL. Studies have shown that the factors affecting EVL efficacy include the following. (1) Variceal diameter > 1.0 cm, at which point the ligature ring is unable to tie the entire varicose vein, and the ligature ring often falls off, resulting in fatal bleeding. (2) Ligation techniques that directly affect the efficacy include: correct positioning of varicose veins, adequate attachment of sucking, making the venous bulb in the transparent cap as full as possible, and avoiding ineffective sucking. This study shows that for varicose vein diameter > 1.5 cm, not completely ligation. For varicose vein diameter < 0.3 cm, the ligation device directly inhaled the esophageal muscle layer into the transparent cap; Then ligation the muscle layer. In this case, the patient experienced long-term postoperative pain and scar stenosis. (3) For varicose veins with severe mucosal erosions or blood blisters, excessive sucking during ligation can sometimes cause severe mucosal damage and induce bleeding, which directly affects the efficacy^27^.

In this study, in the 103 variceal ligations in D1 group (diameter 0.4–1.0 cm), the rate of complete ligation was 95.15% (98/103), it indicates that the ligated vascular root is firm and determined. In 151 variceal ligations in D_2_ group (diameter 1.1–1.5 cm), the rate of complete ligation was 31.13% (47/151). However, in 153 variceal ligations in D_3_ group (diameter 1.6–2.0 cm), the rate of complete ligation was 0%. Thus, for varices with diameter 0.4–1.0 cm, the ligation effect was most complete, and the success rate was the highest, while the ligation failure rate was highest for varices > 1.6 cm in diameter. Therefore, it is particularly important to measure the diameter of esophageal varices on an endoscopic scale^27,28^. This can better apply LDRf typing to guide endoscopic therapy, reduce the risk of endoscopic variceal hemorrhage, and improve the success rate of hemostasis.

In this study, the ligation diameter of esophageal varices in pigs was < 1.5 cm, and this experimental results are consistent with those reported in previous literatures. EVL has rapid eradication of varicose veins and few complications, but the recurrence rate of varicose veins is high. EVL can block the bleeding collateral of the left gastric vein, esophageal vein and vena cava, but after the blood flow of the esophageal vein is blocked, the gastric coronary vein and the perigastric venous plexus have vascular dilation, increased blood flow, and increased recurrence rate over time, so repeated ligation and consolidation therapy are often required^21–24^.

When cirrhosis portal hypertension occurs, the pressure in esophageal varices increases, causing venous dilation, diameter increase, venous wall thinning, and venous wall tension increase. When the critical value is reached, the venous wall rupture bleeding^29–31^. Our previous experimental studies have confirmed that the esophageal variceal pressure is a major factor affecting ligation^32^.

To date, there have been published literature on life threatening massive bleeding due to ulceration following variceal banding and early spontaneous slippage of rubber bands^33–35^, especially 5 to 10 days after the procedure when the O-ring detaches.However, we will be unable to predict the presence of an ulceration with a residual vein at its base and to evaluate the thrombosis of that vein after EVL until detachment of the O-ring^36^. There are other literature reports that percutaneous transhepatic obliteration (Percutaneous transhepatic obliteration, PTO) is one of the useful rescue procedures for life-threatening bleeding after EVL for severe esophagogastric varices^37^. or additional sclerotherapy immediately after EVL may be required to prevent this fatal complication. Thus ,further studies will be needed in order to identify the specific risk factors for potentially fatal complications of this type^38^.

In this experimental model, one limitation of the present study was that we used normal vessels dilated that do not correspond to the natural esophageal varices formation since the varicose veins vessels are not normal because their varicose veins angiogenesis happen as a vessel slowly becomes blocked, and the lack of the present method is that could not be compared to the natural esophageal varices produced by cirrhosis. I think although interfering in the results for guiding role of esophageal variceal diameter in treatment of endoscopic ligation, but this experiment is based on the previous research project "Esophageal variceal pressure influence on the effect of ligation"^31^, and variceal veins of different diameters were simulated with the esophagus of animal pigs to perform endoscopic ligation experiments, and further verified the guiding effect of variceal vein diameter on endoscopic ligation in LDRf typing. So, I believe this animal simulation experiment has preliminarily verified the guiding effect of varicose diameter in LDRf typing on endoscopic ligation. However, a large number of clinical studies are needed to verify the guiding role of LDRf typing. Certainly, the results of this animal simulation experiment maybe it would be more efficient as a simulation for endoscopic ligation training.

Therefore, multi-factor experiments are needed, and measuring the diameter and pressure of esophageal varices has important clinical significance for evaluating the risk of bleeding and its control effect.

### Supplementary Information

Supplementary Table 1.

## Data Availability

The majority of dataset analysed during the current study is available in Supplementary Table 1, while other raw datasets generated during this study are available form the corresponding author (Zhiqun Li) on reasonable request.
