# Supplementary material for: Guiding role of esophageal variceal diameter in treatment of endoscopic ligation: an animal experimental study
Source: Sci Rep. 2024 Feb 16;14:3929. doi: 10.1038/s41598-024-53752-3 (PMC10873287; doi:10.1038/s41598-024-53752-3)
Supplement: Supplementary file 1 — Supplementary Table 1. [file 41598_2024_53752_MOESM1_ESM.doc]

**Table 1**. Linear χ^2^ trend test of the bidirectional ordered grouping data

The ratio of complete ligations for the swine esophageal variceal difference diamete

| variceal diamete | total of ligation | total of successful ligation | Percentage of ligation | the ratio of complete ligations |
| --- | --- | --- | --- | --- |
| D_1_, 0.4–1.0 cm | 103 | 98 | 98/103 | 95.15%, |
| D_2_, 1.1–1.5 cm | 151 | 47 | 47/151 | 31.13% |
| D_3_, 1.6–2.0 cm | 153 | 0 | 0/153 | 0% |

**Note:** Linear trend χ^2^=335.7222, *P*=0.0000. The grouping by vein diameter: D_1_ group, 0.4–1.0 cm; D_2_ group, 1.1–1.5 cm; D_3_ group, 1.6–2.0 cm.
